# Harmonized One Health Trans-Species and Community Surveillance for Tackling Antibacterial Resistance in India: Protocol for a Mixed Methods Study

**DOI:** 10.2196/23241

**Published:** 2020-10-30

**Authors:** HOTSTAR-India Study Group, Manoja Kumar Das, Ashoka Mahapatra, Basanti Pathi, Rajashree Panigrahy, Swetalona Pattnaik, Sudhansu Shekhar Mishra, Samarendra Mahapatro, Priyabrat Swain, Jayakrushna Das, Shikha Dixit, Satya Narayan Sahoo, Rakesh N Pillai

**Affiliations:** 1 The INCLEN Trust International New Delhi India; 2 Department of Public Health The INCLEN Trust International New Delhi India; 3 Department of Microbiology All India Institute of Medical Sciences Bhubaneswar, Odisha India; 4 Department of Microbiology Kalinga Institute of Medical Sciences Bhubaneswar, Odisha India; 5 Department of Microbiology Institute of Medical Sciences and SUM Hospital Bhubaneswar, Odisha India; 6 Department of Microbiology Hi-Tech Medical College Bhubaneswar, Odisha India; 7 Fish Health Management Division Central Institute of Freshwater Aquaculture (ICAR) Bhubaneswar, Odisha India; 8 Department of Pediatrics All India Institute of Medical Sciences Bhubaneswar, Odisha India; 9 Department of Veterinary Surgery College of Veterinary Science and Animal Husbandry (OUAT) Bhubaneswar, Odisha India; 10 Department of Environmental Health The INCLEN Trust International New Delhi India

**Keywords:** bacterial infection, antibiotics resistance, sentinel surveillance, drug prescriptions, One Health, India

## Abstract

**Background:**

India has the largest burden of drug‑resistant organisms compared with other countries around the world, including multiresistant and extremely drug‑resistant tuberculosis and resistant Gram‑negative and Gram‑positive bacteria. Antibiotic resistant bacteria are found in all living hosts and in the environment and move between hosts and ecosystems. An intricate interplay of infections, exposure to antibiotics, and disinfectants at individual and community levels among humans, animals, birds, and fishes triggers evolution and spread of resistance. The *One Health* framework proposes addressing antibiotic resistance as a complex multidisciplinary problem. However, the evidence base in the Indian context is limited.

**Objective:**

This multisectoral, trans-species surveillance project aims to document the infection and resistance patterns of 7 resistant-priority bacteria and the risk factors for resistance following the One Health framework and geospatial epidemiology.

**Methods:**

This hospital- and community-based surveillance adopts a cross-sectional design with mixed methodology (quantitative, qualitative, and spatial) data collection. This study is being conducted at 6 microbiology laboratories and communities in Khurda district, Odisha, India. The laboratory surveillance collects data on bacteria isolates from different hosts and their resistance patterns. The hosts for infection surveillance include humans, animals (livestock, food chain, and pet animals), birds (poultry), and freshwater fishes (not crustaceans). For eligible patients, animals, birds and fishes, detailed data from their households or farms on health care seeking (for animals, birds and fishes, the illness, and care seeking of the caretakers), antibiotic use, disinfection practices, and neighborhood exposure to infection risks will be collected. Antibiotic prescription and use patterns at hospitals and clinics, and therapeutic and nontherapeutic antibiotic and disinfectant use in farms will also be collected. Interviews with key informants from animal breeding, agriculture, and food processing will explore the perceptions, attitudes, and practices related to antibiotic use. The data analysis will follow quantitative (descriptive and analytical), qualitative, and geospatial epidemiology principles.

**Results:**

The study was funded in May 2019 and approved by Institute Ethics Committees in March 2019. The data collection started in September 2019 and shall continue till March 2021. As of June 2020, data for 56 humans, 30 animals and birds, and fishes from 10 ponds have been collected. Data analysis is yet to be done.

**Conclusions:**

This study will inform about the bacterial infection and resistance epidemiology among different hosts, the risk factors for infection, and resistance transmission. In addition, it will identify the potential triggers and levers for further exploration and action.

**International Registered Report Identifier (IRRID):**

DERR1-10.2196/23241

## Introduction

The last century has witnessed a significant reduction in infectious disease mortality and morbidity with the use of antimicrobials. Antimicrobial resistance (AMR), especially antibiotic resistance (ABR), poses a major threat to clinical medicine and public health. The development of new antimicrobials and antibiotics is becoming increasingly difficult and is unable to match the pace of emergence of resistance. It is estimated that AMR-attributable deaths shall rise from 700,000 in 2014 to 10 million by 2050, with US $100 trillion lost output [1]. India is the top contributor toward global morbidity and mortality. India also carries the largest burden of drug‑resistant organisms worldwide, including multiresistant and extremely drug-resistant mycobacteria and resistant Gram‑negative and Gram‑positive bacteria. In India, approximately 60,000 newborns die from resistant bacterial infections [2]. It is projected that over 2 million Indians will die because of AMR by 2050 [3]. Infection with methicillin-resistant *Staphylococcus aureus* (MRSA) and methicillin-sensitive *S aureus* (MSSA) increased the risk of death by 5.6 times and 2.7 times, respectively [4]. The attributable risk of death with MRSA was double that of MSSA by 90 days [4]. Infection with resistant *Escherichia coli* and *S aureus* increased mortality risk by 1.8 to 2.5 times at 30 days [5]. Antimicrobial usage has enhanced animal and fish production globally, paralleling the demand. AMR is a major threat to food safety, food security, and socioeconomics of millions of farming communities.

Resistant bacteria are found in humans, animals, birds, aquatics, plants, and the environment (water, soil, and air), and they move between hosts and ecosystems [6]. In India, >70% of *Acinetobacter baumannii*, *E coli*, and *Klebsiella pneumoniae* and >50% of *Pseudomonas aeruginosa* were resistant to broad-spectrum antibiotics (fluoroquinolones and third-generation cephalosporins) [7]. Extended-spectrum beta-lactamase (ESBL)–producing *E coli* strains from chickens and multidrug-resistant *Salmonellae* species have been reported in India [8-12]. In New Delhi, metallo-β-lactamases (NDM-1, *superbug*), ESBL-producing Gram-negative bacteria, and vancomycin-resistant *S aureus* (VRSA) have been reported in milk from cows with mastitis [13,14]. ESBL-producing *Enterobacteriaceae* in tilapia fishes has been reported from urban water bodies and resistant Vibrios from shellfishes has been reported in the market [15,16]. Resistant bacteria and genes have also been isolated from hospital wastewater, sewage, rivers, surface water, and groundwater in India [17-20].

Although ABR and AMR emerges naturally, antibiotic consumption or usage in humans, animals, and agriculture, environmental waste contamination, sanitation, and infection control practices are the potential drivers for increase in ABR and AMR [1,7]. India’s antibiotic consumption (absolute and percentage increase) is highest globally. Between 2000 and 2015, India’s gross antibiotic consumption increased by 103% (3.2-6.5 billion defined daily doses [DDDs]) and antibiotic consumption rate increased by 63% (8.2-13.6 DDDs per 1000 inhabitants) [21]. The prescription behavior, fixed dose combinations, social pressures, and market influences are some of the factors [22]. In 2010, India was the fifth largest consumer of antibiotics in food animals (poultry, pigs, and cattle) and will become the fourth largest consumer of antibiotics in food animals by 2030 [23]. Approximately four-fifth of the antibiotics used in animals are growth promoters [24]. Approximately 40% of the chicken samples in India had high concentrations of antibiotics [25]. Antibiotic residues have been documented in animal milk [24,26]. India is a hot spot for antibiotic use in food animals, with a use of 30 kg per km^2^, which will grow by 312% by 2030 [23]. The global consumption of antibiotics in animals is estimated to be twice that of humans [27].

Surveillance is an essential tool to document and monitor the ABR and risk factors and appropriately inform policies, infection control, and prevention responses at local, national, and global levels. The Global AMR Surveillance System by the World Health Organization targets 8 bacterial species (*Acinetobacter spp., E coli, K pneumoniae, Salmonella, S aureus, Streptococcus pneumoniae, Shigella spp.,* and *Neisseria gonorrhoeae)* [6]. The Antimicrobial Resistance Surveillance Network coordinated by the Indian Council of Medical Research includes 9 types of bacteria (*E coli, K pneumoniae, Enterobacter spp., A baumannii, P aeruginosa, Salmonella spp., S pneumoniae, S aureus, and Enterococcus spp.*) [28]. These surveillance efforts are primarily targeted at human infections. Studies involving animals and environments have targeted isolation of resistant bacteria, resistance genes, and molecular characterization without crosslinking the hosts and their ecosystems [7].

The *One Health* approach attempts to address complex multidisciplinary problems through designing and implementing programs, framing policies and legislation, and conducting research where multiple sectors converge and collaborate for achieving better outcomes in public health, animal or bird or aquatics health, and environmental settings. The *One Health* approach has been advocated in infectious diseases including zoonoses, food safety, and AMR or ABR, considering the interdependence of human, animal, and environmental factors and determinants for emergence of resistance [29]. There is paucity of data from India on *One Health* surveillance. An integrated surveillance system considering data from humans, animals, food, and the environment and antibiotic usage or consumption for humans and animals appears to be critical.

As part of the *Grand Challenge India on Antimicrobial Resistance* program, this district-based ecological surveillance in India attempts to document the resistance pattern of 7 index bacteria isolated from multiple hosts, including humans, animals (livestock, food chain, and pets), birds (food chain birds such as chicken), and freshwater fishes, correlate with their exposure to antibiotics, disinfectants, and other risk factors at individual, household or farm, and community levels, and analyze the data applying geospatial epidemiology.

**Figure 1:**
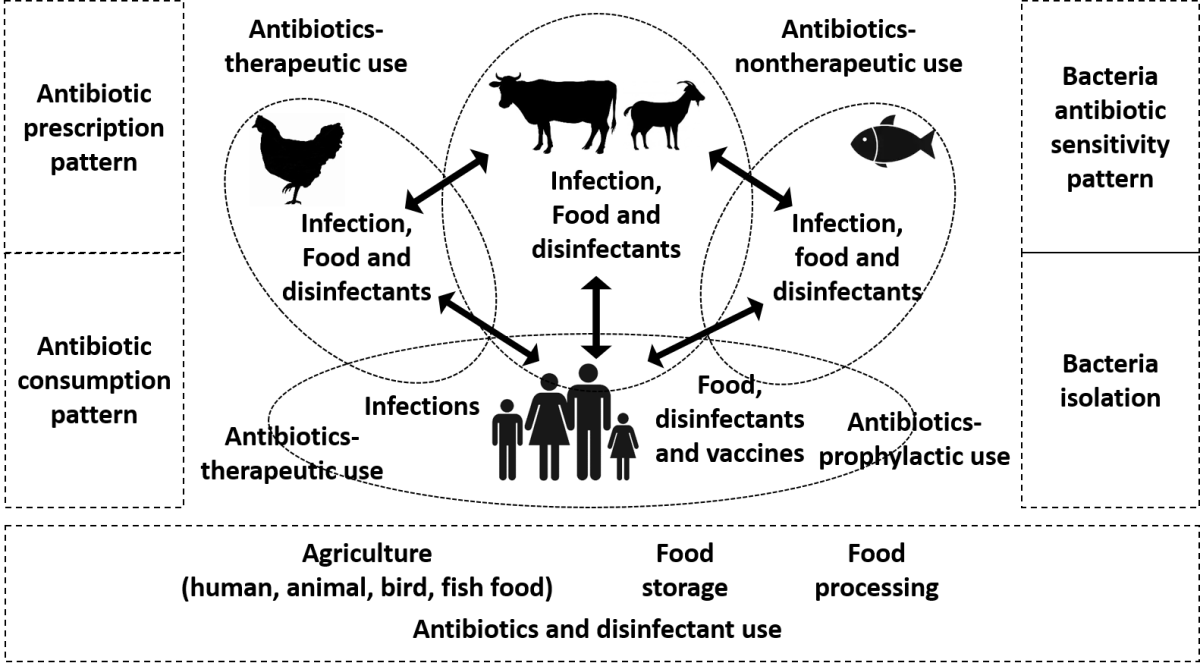
The proposed conceptual model of multihost surveillance for bacterial infections and antibacterial resistance.

An intricate interplay of infections, exposure to antibiotics, and disinfectants at individual and community levels among humans, animals, birds, and fishes triggers the evolution and spread of ABR. The directions of ABR transmission across these species are unclear and probably multidirectional. We hypothesize that concurrent surveillance for index bacterial infections and ABR patterns, exposure to antibiotics and disinfectants, and relevant risk factors for different hosts and environments will improve the knowledge base. The application of multidimensional geospatial epidemiology analysis will inform about the interlinkages between exposures and ecosystems. This multisectoral, trans-species surveillance follows the *One Health* approach and includes 7 priority antibiotic-resistant bacteria: *A baumannii*, *P aeruginosa*, Enterobacteriaceae, *E coli*, *K pneumoniae, S aureus*, *Enterococcus faecium*, and *Salmonellae spp* [30]. Figure 1 shows the conceptual model for the current surveillance.

## Methods

### Objectives

This surveillance aims to (1) document the pattern of infections because of the 7 index bacteria in humans, animals (including livestock, food chain, and pets), birds (food chain birds such as chicken), and freshwater fishes sharing the same environment and their resistance patterns, (2) document the risk factors for ABR at the individual and community level related to health, antibiotic consumption, and antibiotic usage in food animal breeding and agriculture, and (3) apply geospatial epidemiology analytical methodology to improve the understanding of bacterial infections and ABR.

This study aims to (1) conduct surveillance for infection with the index bacteria under study and ABR patterns in humans, animals (including livestock, food chain, and pets), birds (food chain birds such as chicken), and freshwater fishes over 1 year; (2) document the potential factors at the individual, household, or habitation or farm level related to illnesses, care seeking, antibiotic usage, and disinfection that influence resistance among the index bacteria under study, (3) document antibiotic prescription and antibiotic sales patterns for humans (therapeutic and nontherapeutic antibiotic usage in animals including livestock, food chain, and pets), birds (food chain birds such as chicken), and freshwater fishes and agriculture and food processing, and (4) apply multidimensional geospatial epidemiology analysis to generate epidemiological patterns of bacterial infections and ABR and the linkages with the various potential risk factors under study.

### Study Design

This study combines surveillance and cross-sectional design with mixed methodology (quantitative and qualitative) for data collection.

### Study Setting

The study is being implemented at 4 medical college hospitals, 1 veterinary college hospital, and the fishery research institute located in Khurda district, including Bhubaneswar city. The fish samples are being collected from the farming sites in Khurda and the neighboring districts (fish supplying zone). The study is recruiting participants from Khurda district for data collection.

### Study Participants

The study includes 3 categories of participants:

1. Hosts with infections: humans, animals (including livestock, food chain, and pets), birds (food chain birds such as chicken), freshwater fishes (excluding crustaceans) with any of the 7 index bacteria isolates.
2. Patients attending outpatient clinics: humans and animal or birds attended by doctors for prescription audits.
3. Stakeholders for in-depth interviews (IDIs).

The study also involves data collection from 3 types of facilities: (1) microbiology laboratories, (2) chemists and drug distributors for humans and veterinary medicines, and (3) fish farming sites. Table 1 details the categories and numbers of participants.

**Table 1:** The facilities and study participants for data collection under each category.

Serial no. and participant and facility category				Number	
**A: Study participants**					
	**A1: Hosts with infections**				N/A^a^
			A1.1: Humans with positive isolates^b^; (newborns [n=25-30]; children >1 month to 5 years [n=25-30]; and >5 years including adults [n=50-60])		100-120
			A1.2: Animals (including birds) with positive isolates^c^; (animals [n=30-35] and birds [n=20-25])		50-60
			A1.3: Fishes with positive isolates^d^; (fish farms [n=20]; 5-6 fishes each weighing >100 grams per farm)		20
	**A2:** **Patients attending out-patient clinics**				N/A
		**A2.1: Human patients for prescription audit (for antibiotics)**			
			Patients (400 per doctor; 100 every quarter)		6000
			Doctors (for human patients) for prescription audit^e^; (disciplines: medicine [n=5], pediatrics [n=5], and surgery [n=5])		15
		**A2.2: Veterinary patients for prescription audit (for antibiotics)**			
			Animals or birds (100 per doctor; 25 every quarter)		800-1000
			Veterinary doctors^e^		8-10
	**A3:** **Stakeholders for in-depth interview**				50
		Farmers (food and vegetable)			20
		Agriculture stockists			5
		Food animal breeders			5
		Poultry breeders			5
		Fish breeders or farmer			5
		Animal food processors and distributors			10
**B: Study facilities**					
	**B1:** **Microbiology laboratories at the participating institutes**				6
		Medical microbiology laboratories			4
		Veterinary microbiology laboratory			1
		Fish microbiology laboratory			1
	**B2: Chemists and drug distributors for humans and animals**				N/A
		**Medical chemists**			12
			Medical college pharmacy (n=4)		4
			Other hospital pharmacy (n=4)		4
			General chemist and distributor (n=4)		4
		**Veterinary chemists**			4
			Near the veterinary college		1
			Other veterinary chemist		3
	**B3:** **Fish farming sites (for antibiotic usage)**				4-5
		Quarterly audit (4 per farm; once every quarter)			16-20

^a^N/A: not applicable.

^b^The patients with positive growth for any one of the 7 index bacteria from any of these samples: blood, urine, stool, pus, sputum, and other sterile body fluid such as cerebrospinal fluid, pleural fluid, and peritoneal fluid.

^c^The animal and bird with positive growth for any one of the 7 index bacteria from any of these samples: blood, urine, pus, stool, other body parts, and milk.

^d^Fish with positive growth for any one of the 7 index bacteria from gut and gill samples.

^e^The prescription audit includes consecutive new patients (not follow-up patients) seen by the respective doctor.

### Selection of Participants

The various study participants and stakeholders shall be selected following strategy.

#### Humans With Positive Isolates

The eligibility criteria included (1) patients from Khurda district, (2) admitted to inpatient departments of the 4 medical college hospitals, and (3) positive culture growth for any index bacteria from samples collected within 48 hours of hospitalization shall be eligible. Of these eligible patients, we shall randomly select according to the age strata (newborns, >1 month to <5 years, and >5 years), type of bacteria, and departments to obtain representative distribution.

#### Animals With Positive Isolates

The eligibility criteria included (1) animal (including livestock, food chain, and pets) and birds (food chain birds such as chicken) with any infection from Khurda district attending the veterinary college hospital, (2) with positive culture growth for any index bacteria from samples, and (3) inpatient samples collected within 48 hours of hospitalization or outpatient samples collected from fresh patients or fresh samples collected from animals or birds in the farms. Of these eligible patients, we shall randomly select according to the animal or bird type; livestock, pet, or food chain animals and birds; and the specimen types to obtain a representative distribution.

#### Fish Farms With Positive Isolates

The eligibility criteria included (1) freshwater fish farms from Khurda or surrounding districts supplying to Khurda district and (2) with positive culture growth for any index bacteria from samples.

#### Doctors (for Human Patients) for Prescription Audits

The doctors shall be identified from the 4 hospitals (1 per discipline) and from other hospitals or clinics in the Bhubaneswar area. These doctors shall be informed about the activity and consent shall be obtained.

#### Veterinary Doctors for Prescription Audit

The doctors shall be identified from the veterinary hospital and other clinics in the Bhubaneswar area. These doctors will be informed about the activity and consent will be obtained.

#### Patients for Prescription Audit

The patients (humans and animals or birds) attending the selected doctors should be eligible. Patients attending for fresh illness (not follow-up visits) will be approached for consent and data collection.

### Data Collection

Table 2 shows the data to be collected for different components and study participants. The data collection for different hosts is detailed below and in Table 2.

**Table 2:** The data components to be collected from various study participants.

Serial no. and category					Data components to be collected		Frequency	
**A: Study participants**								
	**A1: Hosts with infections**							
		A1.1		Humans with positive isolates		Sociodemography and occupation Illnesses, care seeking, and antibiotic usage Sanitation, waste handling, and disinfection practices and animals or birds exposure Household location and environmental risk factors (GPS)		Target: 100-120 10-12 per month Once for each participant
		A1.2		Animals with positive isolates		Demography (types and number of animals or birds, location, and farming period) Illnesses, care seeking, antibiotic usage, and outcome Sanitation, waste handling, and disinfection practices Feeding and nontherapeutic antibiotic usage Caretaker’s illness and antibiotic usage Farm location and environmental risk factors (GPS)		Target: 50-60 5 per month Once for each animal or bird
		A1.3		Fishes with positive isolates		Demography (species, farm address, and farming period) Any illness, antibiotics used, and outcome Sanitation, waste handling, and disinfection practices Feeding and nontherapeutic antibiotic usage, pesticides, and disinfectant usage Caretaker’s illness and antibiotic usage Farm (habitation for domesticated or nonfarm animals or birds) location and environmental risk factors (GPS)		Target: 20 farms 5-6 farms per quarter 5-6 fishes per farm Once for each farm
	**A2: Patients attending outpatient clinics**							
		A2.1		Human patients for prescription audit		Age, gender, diagnosis, and medicines prescribed Doctor attended		Once for each patient 8-10 new prescriptions per week for specific doctor
		A2.2		Veterinary patients for prescription audit		Animal or bird type, diagnosis, and medicines prescribed Doctor attended		Once for each animal or bird 8 new prescriptions per month for specific doctor
		A3		Stakeholders for in-depth interview		Perceptions, knowledge, attitude, practices, and barriers related to antibiotic usage in agriculture, animal breeding, food industry and potential influence on resistance		Once for each participant Interviews once during study period
**B: Study facilities**								
	B1		Human and animal or bird microbiology laboratories		Number of samples received, types of samples (body part or fluid), number of samples with positive bacteria growth, number of samples with positive index bacteria growth, and antibiotic sensitivity		Daily screening	
	B2		Fish microbiology laboratory		Number of samples collected and processed, types of samples (body part), number of samples with positive bacteria growth, number of samples with positive index bacteria growth, and antibiotic sensitivity		Periodic, when sample collected or processed	
	B3		Chemists and drug distributors		For humans and animals: Volume of antibiotics sold		Monthly	
	B4		Fish farming sites		Antibiotic usage, pesticides, and disinfectant usage on quarterly basis		Quarterly	

#### Infections in Humans

##### Surveillance of Bacterial Infections

At the 4 medical college hospitals, daily surveillance will be conducted to identify any positive index bacteria isolates from the samples of the hospitalized patients. For the positive index bacteria isolates, information about antibiotic sensitivity; dates of sample collection and admission; and patient information including diagnosis, antibiotics used, outcome, and basic demography (age and gender) will be documented. Among these patients with positive bacteria isolates, eligible patients (as per the eligibility criteria defined above) will be identified from the admission registers for detailed data collection at the household or farm level.

##### Detailed Individual Data Collection for the Humans With Positive Bacteria Isolates

Of the eligible patients, approximately 120 patients (10-12 patients every month) will be randomly selected. These selected patients (and family members) will be contacted after the discharge or death of the patient to schedule the home visit. During home visits, informed written consent will be obtained, followed by data collection using Case Record Forms (CRFs).

#### Infections in Animals and Birds

##### Surveillance of Bacterial Infections

For the veterinary college hospital, daily surveillance will be conducted to identify any positive index bacteria isolates from the samples of the animal or bird patients attending the hospital or those that were admitted. For the positive index bacteria isolates, information about antibiotic sensitivity, diagnosis, antibiotic use, outcome, and animal or bird type will be documented. Among these animal or bird patients with positive bacteria isolates, eligible animals or birds will be identified from the records for detailed data collection at the household or farm level.

##### Detailed Individual Data Collection for the Animals and Birds With Positive Bacteria Isolates

Of the eligible animal or bird patients, approximately 60 patients (5 patients every month) were randomly selected. For these selected animals or birds, their owners or caretakers will be contacted for scheduling household or farm visits. During household or farm visits, informed written consent from the owner or caretaker will be obtained, followed by data collection using the CRF.

##### Fish Farms With Positive Isolates

For the freshwater fish farms with positive isolates from fishes (not crustaceans), the owners or caretakers shall be contacted and farm visits shall be made for informed written consent followed by data collection using the CRF.

##### Antibiotic Sales

From the identified chemists and drug distributors for humans, animals, and birds at the hospitals and outside, the data on antibiotic procurement or indent and sales shall be collected on a monthly or quarterly basis. The list shall include oral (tablets, capsules, and syrups) and injectable forms for the different types of antibiotics.

##### Antibiotic Usage at Fish Farming Sites

For the fish farming sites, quarterly visits will be made to collect information on the use of different antibiotics or disinfectants or chemicals or growth promoters (therapeutic or nontherapeutic) and their quantity.

##### Doctors for Prescription Audits

For the doctors (human and veterinary), the hospital or clinic and specialty will be documented.

##### Patients for Prescription Audits

The consecutive patients (and their parents) attending the outpatient department of the identified doctors with a new illness will be approached at exit (consultation completed) for participation. For patients who consent, the age, sex, diagnosis, and medications prescribed will be captured. Similarly, for the animal or bird patients attending the veterinary doctors, the owners or caretakers will be approached for consent to participate. For the animals and birds, the species type, diagnosis, and medicines prescribed will be captured.

##### Geospatial Data Collection

The precise location data (latitude and longitude) for the households or habitations or farms of the recruited human participants, animals and birds, and fish farming sites with positive bacteria isolates shall be collected using a GPS device (Garmin Montana 680, Garmin). For these participants, neighborhood mapping covering a 100-m radius around their locations will capture the potential risk factors for infection (garbage dump, wastewater, animal or poultry farm, egg or meat vending, hospital or clinic, chemist, hotel or food selling, industry, etc.) with their GPS positions. The technologies to be used for geospatial mapping include GPS, geotagging, and geographic information systems. The GPS data points collected shall be mapped on the state and district satellite map.

##### Stakeholders for IDIs

The stakeholders or key informants shall be identified purposively considering the profession (agriculturist, stockiest, breeder, food processing, and distribution) and geography to suit the objectives. All IDIs shall be conducted at a convenient place for the participant after obtaining informed consent and the conversation shall be digitally recorded with consent. The IDI will focus on exploring the perceptions, knowledge, attitude, practices, and barriers related to antibiotic use in agriculture, animal breeding and food industry and the potential influence on resistance. The IDIs conducted in local language shall be transcribed verbatim and translated to English. Quality checks of transcripts and translation will be performed for 25% of the audio recordings by another member.

**Figure 2:**
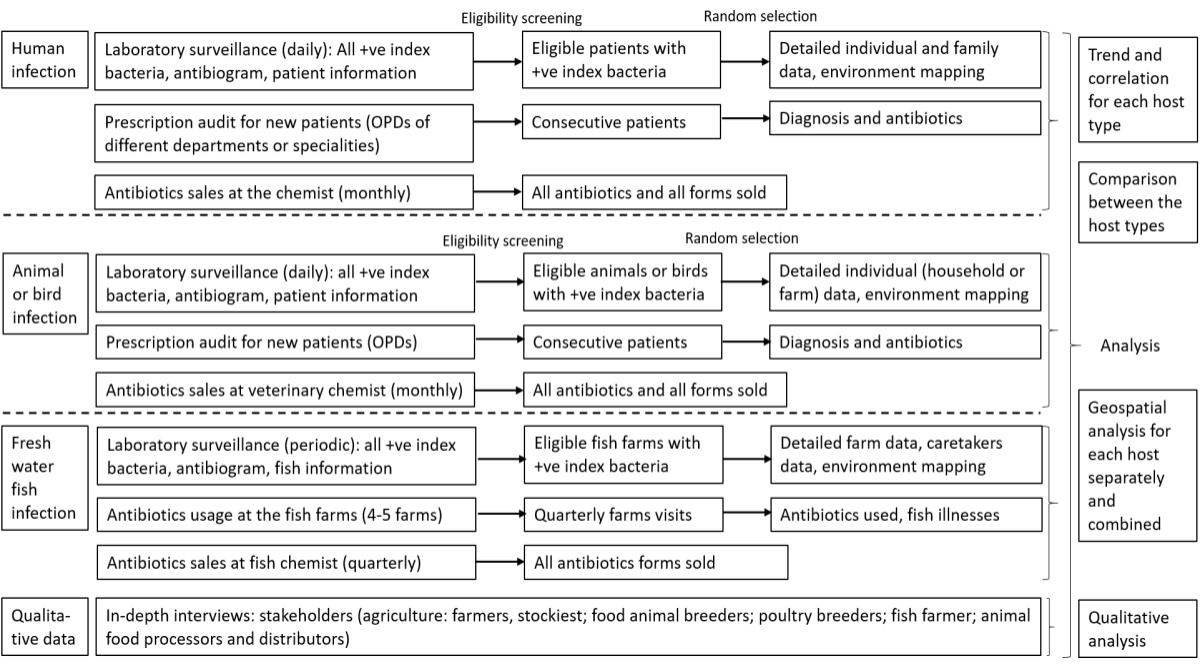
The workflow of surveillance data collection targeted at different hosts. OPDs: outpatient departments.

The surveillance and data collection workflow are shown in Figure 2.

### Data Management

The surveillance and quantitative data shall be collected using customized software installed on tablets (developed using open source platforms: Android; PHP, the PHP Group; and MySQL, Oracle Corporation) and uploaded to the server through a mobile network. The data collection and transmission process shall have encryption and security measures. The qualitative data shall be collected using IDI guides on paper, followed by transcription, translation, and data entry. All electronic data will be stored in a secured server with multilayered security and daily backup. The investigators and authorized research staff have data access.

### Data Analysis

**Figure 3:**
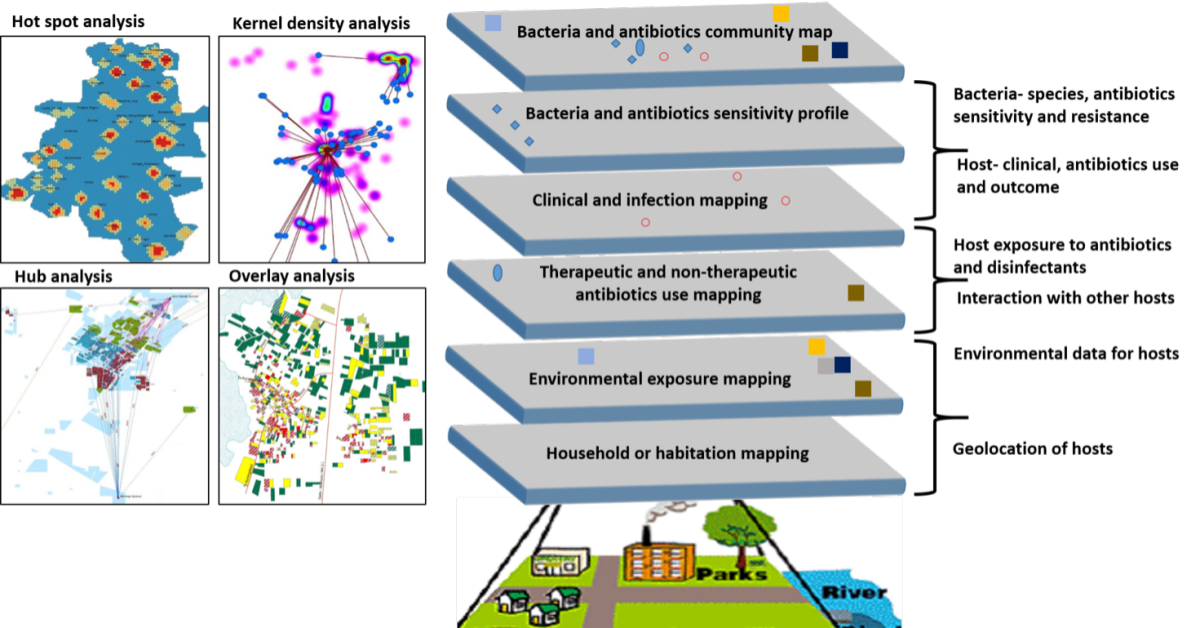
Proposed geospatial epidemiology analysis outputs.

The quantitative data are expressed as means (with standard deviations), medians (with interquartile ranges), and proportions using descriptive statistics. The data for different groups will be compared using *t* tests, chi-square tests, Mann-Whitney test, and Kruskal-Wallis test, as appropriate. The Jonckheere-Terpstra test will be used to assess the monthly trend of bacteria isolates (proportions), the ABR pattern for antibiotics, antibiotic prescription, antibiotic sales, and statistical significance. The Pearson correlation coefficient will be used to examine the relationship between antibiotic prescription and sales and ABR rates. The qualitative data will be analyzed by content analysis as per the domains identified and follow processes: free listing, coding, axial coding, and cross tabulation. The spatial data will be analyzed using geospatial epidemiology principles, including point pattern analysis (clustering and density), kernel density map (hot spots and catchment), hub analysis (common exposures and catchment area), and overlay analysis (exposures or risk factors layering and spatial correlation; Figure 3). The GPS and geospatial data will be analyzed using ArcGIS, QGIS, and Global Mapper 2.0.

### Validity and Reliability

Uniform laboratory processing, antibiotic sensitivity testing, and interlaboratory comparison will be practiced. Monthly teleconference and quarterly site visits will focus on protocol adherence and data validation. The data collected will undergo consistency and range checks by the data management team.

### Ethical Aspects

The study protocol was reviewed and approved by the INCLEN Ethics Committee, New Delhi (Ref: IIEC-056), Institute Ethics Committee, All India Institute of Medical Sciences, Bhubaneswar (Ref: T/EMF/Micro/18/6); Kalinga Institute of Medical Sciences Medical Research Committee (KIMS/R&D/255/2018), Institute Ethics Committee, Institute of Medical Sciences & SUM Hospital (Ref: DMR/IMS.SH/SOA/180167); Institutional Ethics Committee for Human Research, Hi-Tech Medical College & Hospital (Ref: HMCH/IEC/19-004). The participants are being recruited after obtaining written informed consent. Appropriate data confidentiality, storage, and access authorization procedures are being adopted.

## Results

The study was funded in May 2019 and approved by Institute Ethics Committees in March 2019. The data collection started in September 2019 and shall continue till March 2021. As of June 2020, data for 56 humans, 30 animals and birds and fishes from 10 ponds have been collected. The other data collection is also in progress. Data analysis is yet to be done.

## Discussion

### Antibiotics Resistance Problem in India

A scoping report on ABR in India reported that resistance to broad-spectrum antibiotics fluoroquinolones and third-generation cephalosporin was >70% in *A baumannii*, *E coli*, and *K pneumoniae* and >50% in *P aeruginosa* [7]. The isolation of ESBL-producing *E coli* from chickens, multidrug-resistant *Salmonellae* from chicken meat samples, VRSA from cow milk samples, and ESBL-producing *Enterobacteriaceae* from fishes indicate the spread of ABR across all food animals and in the environment. Relatively unregulated and high antibiotic usage in humans and among animals and aquatics in India is worrisome. The heavy use of antibiotics is based on high infectious disease burden and past experiences of mortality and a limited understanding of the impact across sectors. The implementation of the One Health framework in policy and program has been challenging in view of simultaneous and connected evidence from the Indian context. There is a need to understand the drivers for the emergence of ABR and transmission across the host-environment systems for appropriate action.

### Strengths and Limitations

The multihost simultaneous surveillance for the 7 resistant-priority bacteria among humans, animals, birds, and fishes following the One Health framework is a strength of this study. Therapeutic antibiotic usage for therapeutic purposes among these patients and nontherapeutic usage for animal breeding will allow drawing possible linkages with the resistance pattern. The geospatial epidemiology analysis is expected to provide information about the pattern and linkages between the variables. The shorter observation period may not permit inferring any causal association between the various risk factors and antibiotic resistance.

### Conclusions

This study will generate evidence on (1) epidemiology of infections with 7 high-priority bacteria among the different hosts (human, animal, birds, and fishes) and their resistance patterns, (2) the potential risk factors for ABR in the infected hosts at individual and immediate environment levels, (3) therapeutic and nontherapeutic antibiotic usage for humans, animals, fishes, agriculture, and food processing along with the potential triggers, and (4) potential linkages between data from various sources and identification of possible risk factors for ABR across various hosts and their ecosystems. India is critically important for ABR and transmission of multidrug-resistant bacterial species because of high antibiotic consumption, unrestricted therapeutic and nontherapeutic usage across all sectors, and other environmental and behavioral risk triggers. This multisectoral, trans-species surveillance for bacterial infection and ABR using *One Health’s* perspective and geospatial epidemiology techniques will improve our understanding of the pattern and spread of resistance and potentially inform about the potential levers for actions in public health, animal, bird and aquatics health, and environmental settings.

## Appendix

Multimedia Appendix 1 Peer review reports from PMU-BIRAC.

## Abbreviations

ABR: antibiotic resistance
AMR: antimicrobial resistance
CRF: Case Record Form
DDD: defined daily dose
ESBL: extended-spectrum beta-lactamase
IDI: in-depth interview
MRSA: methicillin-resistant Staphylococcus aureus
MSSA: methicillin-sensitive Staphylococcus aureus
NDM: New Delhi metallo-beta-lactamase
OPD: outpatient department
VRSA: vancomycin-resistant Staphylococcus aureus
