# Supplementary material for: Harmonized One Health Trans-Species and Community Surveillance for Tackling Antibacterial Resistance in India: Protocol for a Mixed Methods Study
Source: JMIR Res Protoc. 2020 Oct 30;9(10):e23241. doi: 10.2196/23241 (PMC7665953; doi:10.2196/23241)
Supplement: Multimedia Appendix 1 [file resprot_v9i10e23241_app1.pdf]

Grand Challenges India Antimicrobial Resistance call

Proposal number: BT/AMR0317/05/18

Proposal title: Harmonized One health Trans-species and community Surveillance for Tackling Antibacterial Resistance in India HOT-STAR-India

Submitted by: Manoja Kumar Das, The INCLEN Trust International

Technical Review

The proposal focuses on One Health approach and includes surveillance for infections in humans, animal and birds, and fishes and tries to link the antibiotics usage with resistance. The proposal proposes to use geospatial technology and qualitative methods (interviews) for additional data collection and analysis.

It is a good proposal. The proposal needs clarification on the following:

1. How the species identification and antibiotic sensitivity testing across laboratories be standardised and made comparable?
2. How the geospatial analysis shall be undertaken?

Responses to the comments/queries

We would like to submit the following clarifications and explanations.

1. How the species identification and antibiotic sensitivity testing across laboratories be standardised and made comparable?
   Response: The specimens with positive bacterial growth shall be subjected for species identification as per the recommended guidelines including the gram stain, biochemical tests followed by Vitek 2 cards for gram positive and negative organisms. For the antibiotics sensitivity testing, we shall be following the Kirby-Bauer disc diffusion method using Mueller-Hinton agar and interpretation according to the Clinical and Laboratory Standards Institute (CLSI 2019) guidelines using discs of standard concentration. The isolates shall be also subjected for AST test using the VITEK 2 cards according to the species (gram positive and gram negative). We shall be harmonising the laboratory protocols at these laboratories and develop Standard Operating Procedures for the processes to ensure standardisation and comparability.

2. How the geospatial analysis shall be undertaken?
   Response: We shall be collecting the GPS data (latitude and longitude) for the households/habitations/farms of the humans/animal farms/bird farms/fish farming sites with positive bacterial isolates, the possible risk factors, and the sources of the antibiotics exposure. These GPS coordinates shall be mapped on the district map. We shall use the geospatial epidemiology methods for analysis including point pattern analysis (clustering and density), kernel density map (hot spots and catchment), hub analysis (common exposures and catchment area) and overlay analysis (exposures/risk factors layering & spatial correlation).
